# Jenner’s Legacy: How Edward Jenner’s Smallpox Vaccine Changed Public Health

**DOI:** 10.7759/cureus.68594

**Published:** 2024-09-03

**Authors:** Ansar Ahamed VP, Sonali G Choudhari, Abhay Mudey, Abhishek Joshi

**Affiliations:** 1 Department of Community Medicine, School of Epidemiology and Public Health, Jawaharlal Nehru Medical College, Datta Meghe Institute of Higher Education and Research, Wardha, IND

**Keywords:** smallpox, immunology, preventive medicine, vaccination, historical vignette

## Abstract

Edward Jenner who discovered immunology and the smallpox vaccine conducted a smallpox vaccination test in 1796, which is considered a landmark in the history of modern immunization. This review focuses on describing Jenner and his accomplishments highlighting his discovery as a shift in the approaches toward disease control and prevention as well as the basis for further eradicating smallpox globally. Jenner’s use of cowpox to protect people from smallpox was a revolution from other essential procedures such as variolation. His vaccine demonstrated how immunization could be used to combat diseases, and over the years the idea began to be deployed to other vaccines and other diseases.

Besides the given medical profession, the work of Jenner was relevant to the changes or even enhancements of health policies and health systems globally. His vaccine provided not only the means to arrest a wide disease that could easily eliminate many human beings but also initiated procedures of disease prevention and control. Another important type of immunity, herd immunity, which determines an approach to managing specific diseases in the present, as well as knowledge of the degree of protection provided by the antigen in terms of the overall immune status of a population, was also achieved based on the actions taken after Jenner’s procedure.

As our society searches for vaccines for emergent diseases to date, this review reveals that Jenner’s accomplishments are still relevant. In this article, analyzing Jenner’s approach and the role of society and science at the time, along with the consequences of his work, the reader sees how Jenner changed the world of public health and laid a foundation for today’s vaccination. Such an approach allows considering the changes and evolution of the topic that Jenner studied and contributed to and how people fight infections today.

## Introduction and background

Edward Jenner, the son of the village vicar, was born in Berkeley, Gloucestershire, on May 17, 1749. He was a surgeon and the first one to vaccinate against smallpox. Jenner attended Wotton-under-Edge and Cirencester for his education [1]. During this time, he was immunized against smallpox, which had a long-lasting impact on his overall health. He relocated to London’s St. George’s Hospital in 1770 to finish his medical training under the training of famous inventor and physician John Hunter. Edward’s enthusiasm immediately struck Hunter for dissection and study, as well as his familiarity with the anatomy of both plants and animals. The two men would remain lifelong friends and communicators [1]. At the age of 14, he finished his studies in London and began working as an apprentice to a local surgeon. After returning to Berkeley in 1772, he spent most of his professional career practicing medicine in his birthplace [2]. Jenner married Catherine Kingscote in March 1788; she died of tuberculosis in 1815. He may have first seen her while playing with balloons with a group of other boys. Jenner’s experimental balloon landed on Anthony Kingscote, Catherine’s father’s home in Kingscote Park, Gloucestershire. Their three children were Catherine (1794-1833), Robert Fitzharding (1792-1854), and Edward Robert (1789-1810) [3].

**Figure 1:**
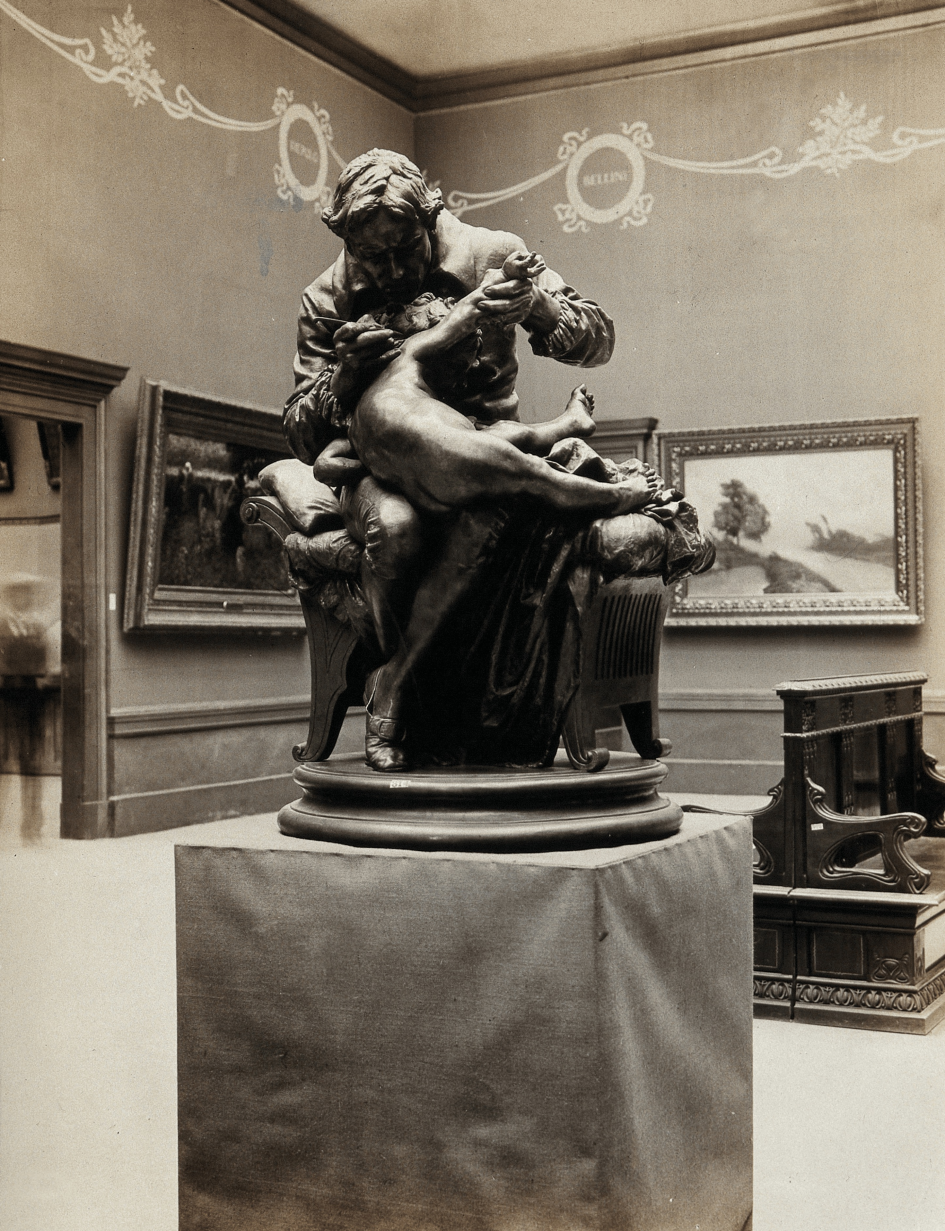
Edward Jenner injecting the vaccine into his son, a sculpture by Giulio Monteverde. Image credit: Edward Jenner, photograph of a sculpture by Giulio Monteverde. Galleria Nationale d’Arte Moderna, Rome, Wellcome collection [5].

Edward Jenner’s extensive research on the efficacy of cowpox immunization against smallpox in the late 1790s, as well as his passionate and unwavering advocacy of vaccination despite opponents’ doubts, laid the groundwork for 19th-century insights into the origins of immunity and the nature of infectious diseases. Jenner initiated the extensive procedure that led to the successful eradication of the smallpox virus in 1980 [4]. Figure 1 depicts Edward Jenner injecting the vaccine into his son, a sculpture by Giulio Monteverde, in 1873, in the Palazzo Bianco, Genoa, Italy [5].

His life narrative continues to serve as motivation for doctors who face an uncertain future due to the adaptation of bacteria and viruses that have not yet been defeated by antibiotics [4]. Smallpox destroyed humanity for countless generations. With gratitude for Edward Jenner’s remarkable endeavor and the subsequent achievements, we no longer need to worry about it. Because of the current explosion in vaccine discoveries, the historical background of immunization is often ignored [6].

Jenner is commonly referred to as the father of immunology in the Western world [2], and more lives than any other man are said to have been saved by his efforts [7]. During Jenner’s time, smallpox killed around 10% of the world’s population, perhaps up to 20% in urban areas where the illness spread rapidly. In 1821, he became Berkeley’s mayor and justice of the peace in addition to serving as King George IV’s physician. He belonged to the Royal Society. His description of the cuckoo’s brood parasitism was among the first by contemporary biologists (this habit was discussed by Aristotle in his History of Animals). In 2002, the BBC named Jenner one of the 100 Greatest Britons [3].

The Fleece Inn parlor in Rodborough, Gloucestershire, served as the meeting place for Jenner and other members of the Fleece Medical Society, also known as the Gloucestershire Medical Society. Members read medical-related papers while eating dinner together. Jenner made comments on cowpox and provided papers on ophthalmia, heart valve disease, and angina pectoris. He was a member of a comparable group that convened in Alveston, close to Bristol [3].

Smallpox was a terrible illness with high death rates and significant scarring in survivors before Jenner’s discovery [8]. In addition to transforming medicine, Jenner’s smallpox vaccine invention launched the immunization program that still saves countless lives today. The impact of Jenner’s contribution goes beyond the disease’s elimination. His approach of testing and recording the vaccine’s effects, along with scientific rigor, became a model for subsequent vaccine development [9]. Jenner’s discovery showed how vaccinations may be used to prevent infectious diseases, which encouraged more studies and developments in immunology [3]. The broad application of vaccinations to fight a variety of infectious diseases, which finally changed international health standards, is clear evidence of his legacy.

## Review

Smallpox: The legacy of a deadly disease

The natural history of smallpox is unknown to prehistoric humans. It is believed to have started around 10,000 BC, at the commencement of agricultural societies, in northern Africa [10]. It is believed that traders from ancient Egypt brought it to India. The earliest documented eras when mummy faces had skin lesions resembling smallpox were the 18th and 20th Egyptian dynasties (1570-1085 BC). Ramses V, the Egyptian pharaoh who reigned from 1156 to 1156 BC, had symptoms of illness on his mummified head [11]. Smallpox cases have been reported continuously by Asian historical cultures. The disease was first detected in China about 1122 BC and is also mentioned in ancient Sanskrit literature from India.

Likely, smallpox was first brought to Europe in the 5th or 7th century, and during the Middle Ages, outbreaks of the disease were common. The disease had a significant impact on Western civilization’s growth. The Antonine plague was a global epidemic that killed some 7 million people in the Roman Empire in AD 108 [12]. The West Indies’ discovery, Arab settlement, and the Crusades all contributed to the disease’s dissemination.

Smallpox was not detected until the arrival of Spanish and Portuguese conquistadors in the New World. The disease wiped out the indigenous people and played a significant role in the collapse of the Aztec and Inca civilizations. Similarly, early immigrants transmitted the sickness to North America’s East Coast, causing the indigenous population to decline as well. One of the earliest instances of biological warfare resulted from the terrible consequences of smallpox [6,13]. Sir Jeffrey Amherst, the British commander in North America during the French-Indian War (1754-1767), proposed intentionally spreading smallpox to reduce the number of American Indians who resisted the British. The slave trade played a role in the smallpox pandemic that ravaged the Americas, as many slaves were transported from parts of Africa where the illness was common.

Smallpox struck people from all social classes. About 400,000 people in Europe perished from smallpox in the 18th century, and one-third of those who survived went blind [10]. Smallpox symptoms, often referred to as the “speckled monster” in 18th-century England, were extremely lethal and frequently manifested abruptly. Around 20-60% of instances resulted in death, leaving the survivors with scars that might change over time. Neonatal case mortality rates increased significantly in the late 1800s, reaching over 80% in London and 98% in Berlin [11]. Smallpox was commonly referred to by the name “variola,” which was first used in 570 AD by Bishop Marius of Avenches, a town near Lausanne, Switzerland. The phrase originates from two Latin words: varius, which means “stained,” and varus, which means “mark on the skin.” To differentiate it from syphilis, which was then known as large pockes, the illness was known in England around the end of the 15th century as little pockes (pocke meaning sac) [11].

Vaccine: The dawn of preventive medicine

Although vaccinations were already widely used in Asian and African medicine, there were still serious hazards involved, such as the potential for recipients to contract the illness and infect others [14]. It was brought back to Britain by Lady Mary Wortley Montagu in 1721 after she saw variations in Istanbul and Johnnie Notions, who successfully created his vaccine and is said to have never lost a patient [15]. The Shetland Isles were the only place where his method was used. According to Voltaire, 20% of people died from smallpox at this time, and 60% of people caught it [16]. Additionally, Voltaire argues that inoculation had been done by the Circassians for eons, and the Turks may have borrowed the method from the Circassians. Daniel Bernoulli examined statistics on smallpox morbidity and mortality in 1766 to prove that vaccination was effective [17].

An English physician named John Fewster discovered in 1768 that a person resistant to cowpox would also be immune to smallpox [18]. Throughout the years after 1770, at least five researchers in England and Germany successfully tested the cowpox vaccine against smallpox in humans (Rendell, Plett 1791, Sevel, Jensen, Jesty 1774) [19]. Jenner’s research illuminated the process by which Benjamin Jesty, a farmer from Dorset, vaccinated his wife and two children against smallpox in 1774 by giving them cowpox during the outbreak. Jenner may have been aware of Jesty’s strategies and accomplishments. In 1780, Jacques Antoine Rabaut-Pommier noted something similar in France [20].

**Figure 2:**
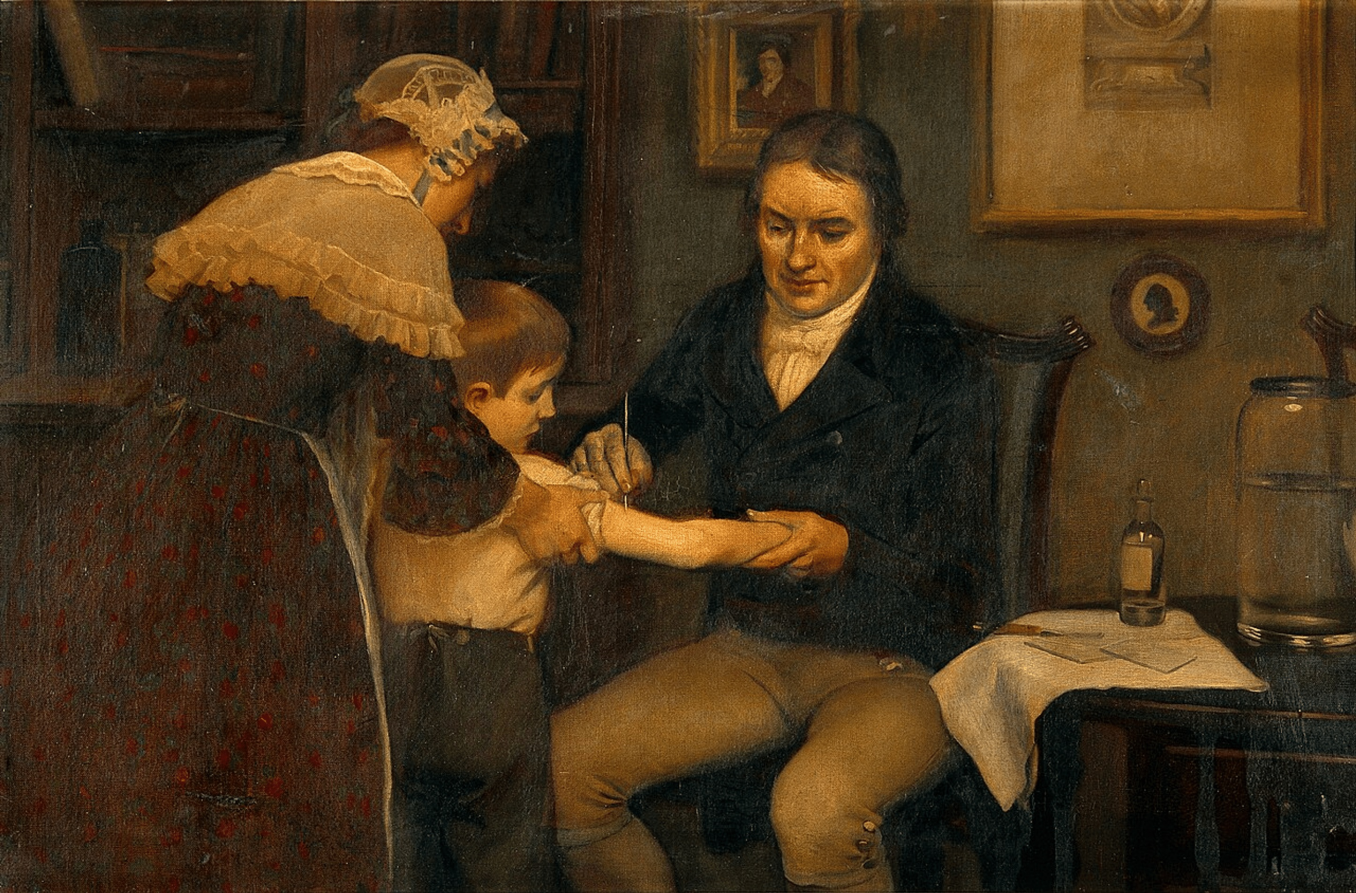
Jenner administering James Phipps his first vaccine. Image credit: The New York Times [20].

Jenner proposed that those with cowpox, a condition related to smallpox but far less virulent, had pus in their blisters that shielded them from smallpox. On May 14, 1796, Jenner’s gardener’s son, James Phipps, aged eight years, was injected to test Jenner’s assertion. After milkmaid Sarah Nelmes got the disease from a cow named Blossom, he extracted pus from blisters on her hands. The Tooting-based St. George’s Medical School library has an exhibit depicting this. Figure 2 depicts Jenner administering James Phipps, an eight-year-old kid, his first vaccine on May 14, 1796 [20].

Jenner gave Phipps injections in both arms that day; there were no major side effects, except a mild temperature and indigestion. Later, he administered a variolous material shot to Phipps, following the then standard immunization protocol. There was no subsequent illness. Again, following the exposure to a volatile material, the child showed no signs of infection. Beyond that, neither Phipps nor the other patients experienced any “breakthrough” experiences. There were no unexpected side effects.

Jenner’s biographer, John Baron, subsequently claimed that, rather than elaborating on his forefathers’ research, Jenner found that cowpox exposure could be used to implant immunity against smallpox by observing the spotless skin of milkmaids. Even though it seems like fiction, people still tell the milkmaids’ story all the time [21,22].

Donald Hopkins claims that the real achievement of Jenner was not protecting a limited number of people from cowpox but rather proving via additional testing that they were immune to smallpox. In addition, he demonstrated that human-to-human transmission of the protective cowpox pus was feasible in addition to cattle-to-human transmission. Jenner’s recommendations were successfully tested on an additional 23 humans.

Jenner’s initial work was never published by the Royal Society, but he sent his research to them nevertheless. He released his findings on the 23 cases after making modifications and conducting additional research, which included his son Robert, who was 11 months old [3]. His research could easily be replicated using contemporary microbiological and microscopic techniques; some of his results were true, but others were not. The medical community considered his findings carefully before accepting his conclusions. Once vaccination became popular, the British government banned variolation in 1840, favoring a free cowpox vaccine instead of the practice of using smallpox to induce immunity [3]. Spanish Expedition to the Balmis (1803-1806): Francisco Javier de Balmis led the expedition, which traveled for three years distributing smallpox vaccinations throughout the Americas, the Philippines, and Macao, China. The journey’s popularity quickly spread throughout Europe and was extensively utilized [23].

After the mission was successful, Jenner wrote: “It is unlikely that philanthropy this extensive and so noble will be found in the annals of history” [24]. Napoleon awarded Jenner a medal while he was at war with Britain and let him return home with two English prisoners of war that he had rescued. Napoleon also made vaccinations a requirement for all of his French soldiers. “It is impossible to refuse anything to one of the greatest benefactors of mankind,” said Napoleon [25]. Because of his ongoing efforts on immunizations, Jenner was unable to continue as a regular physician. With the backing of the King and his associates, he petitioned Parliament and was awarded £10,000 for his vaccination research in 1802. He received an additional £20,000 in 1807 following the Royal College of Physicians’ confirmation of the vaccination’s broad effectiveness [26].

Twilight years

Jenner was a well-known member of the American Philosophical Society, the American Academy of Arts and Sciences, and the Royal Swedish Academy of Sciences, among other scientific groups. The Jennerian Society elected him as its president, whose mission was to use vaccination to end smallpox. After the establishment of the national vaccine establishment, Jenner resigned his directorship. He served as King George IV’s extraordinary physician before becoming Berkeley’s mayor [3].

Research contributions

Following this trial, Jenner carried out additional tests. He published all of his smallpox studies in “An Inquiry into the Causes and Effects of the Variolae Vaccinae; a Disease Discovered in Parts of the Western Counties of England, Particularly Gloucestershire, and Known by the Name of the Cow Pox” in 1798. The findings of further research he published in each of the following two years confirmed his initial notion that cowpox did offer protection against smallpox [1].

Demise

Jenner was found in apoplexy, his right side paralyzed, on January 25, 1823. He appeared to have suffered his second stroke but was unable to recover and died on January 26, 1823, at the age of 73. The Berkeley Church of St. Mary family vault served as his final resting place [7].

Heritage

The World Health Organization proclaimed smallpox to be extinct in 1980. Immunization was a crucial component, but integrated public health initiatives produced the desired outcome. Even though the disease has been declared eradicated, certain pus samples are maintained in labs at the Centers for Disease Control and Prevention in Atlanta, US, and the State Research Center of Virology and Biotechnology VECTOR in Koltsovo, Novosibirsk Oblast, Russia [3].

## Conclusions

One of the outstanding accomplishments in the sphere of public health and medicine was made in the late 1700s by Edward Jenner. Jenner’s invention of the smallpox vaccine paved the way for the growth of immunology as well as demonstrated that science could bring about a positive health impact. Though looked very primitive in today’s world, his techniques explained how vaccinations might reduce the chances of an ailment and even possibly save lives.

Edward Jenner not only contributed significantly toward eradicating smallpox but also left a progressive influence on society. His approach toward immunization led to multiple vaccinations that have since then averted so many illnesses as well as deaths globally. Thus, the story of Jenner is an example, on the one hand, of perseverance, scientific interest, and a compass in adversities and skepticism. This work contributed to the development of present immunization campaigns that protect the public from infectious diseases.

By analyzing Jenner’s life and achievements in detail, we can recognize vaccine’s relevance to the current and potential fight against diseases as well as our health. Exploring the story connected to Jenner, one highlights the potential of science to tackle some of the existing issues and enhance people’s quality of life. The global research community and healthcare practitioners are inspired by his forward-thinking and passion for improving people’s quality of life. It underlines the relevance of his contributions to the never-ending struggle against communicable diseases.
